# The impact of intellectual property protection on the development of digital economy and regional entrepreneurial activity: Evidence from small and medium enterprises

**DOI:** 10.3389/fpsyg.2022.951696

**Published:** 2022-07-22

**Authors:** Hong Chen

**Affiliations:** Shanghai International College of Intellectual Property, Tongji University, Shanghai, China

**Keywords:** intellectual property, open innovation, information communication technology, commercialization performance, entrepreneurial performance

## Abstract

Substantial intellectual property management (IPM) is vital in retaining competitive advantage and managing outbound open innovation (OI), which may enhance an organization’s commercialization and entrepreneurial performance. Thus, the objective of this study was to develop an understanding of the impact of intellectual property protection on the development of the digital economy, regional entrepreneurial activity, and explore how IPM can enhance the entrepreneurial performance (EP) through open innovation (OI) and commercialization performance (CP) in the context of small and medium enterprises (SMEs) in Mainland China. Our study also shows how open innovation model constraints (OIMC) and information communication technology (ICT) enhance the performance. Using the organizational performance theory, we developed our research framework and collected usable data from 530 respondents from the management of SMEs in Mainland China. Data analyses were performed using SPSS, and structural equation modeling was performed using Amos 24 to test the hypothesis. Our results highlighted the significant effect of IPM on OI, CP, and EP. This study suggests various conclusions, stressing the mediating function of CP in improving EP and the direct and indirect effects of OI and CP on EP. This study also emphasizes that business managers need to ensure collaboration among SMEs since it is the best strategy to use each other’s resources, including OI ideas, to improve the EP, and it should be done utilizing ICT.

## Introduction

Small and medium enterprises (SMEs) act as engines that drive economic progress. Current digitalization and globalization have changed the way businesses are operated, even forcing them to elect modern organizational practices to ensure the improved performance of the business (Alper et al., 2022). For any organization, the skills of their workforce, level of knowledge and competency, and the ability to exhibit creativity are possible sources to gain competitive advantages (Fisher and Oberholzer-Gee, 2013). Management of such intellectual capital by a firm can encourage innovation and prevent competition (Greenhalgh and Rogers, 2007). Wang (2021) mentioned that Intellectual property management (IPM) is an essential element of scientific and technological development; it ensures innovation and creativity (Laplume et al., 2014; Uyar et al., 2021; Olubiyi et al., 2022). Countries across the globe are making efforts to promote the establishment and execution of capabilities for IPM to boost their innovation levels (Qayyum et al., 2022; Thakur-Wernz and Wernz, 2022), and to extend the support for improved quality products for export purposes. With continuous economic development in China, its government is vigorously working to implement the IPM strategies with a vision to become an innovative country and build China into an intellectual property power. Different studies were conducted to understand the digital economy prosperity and growth of the target market, but most of them were based on the entrepreneurial performance with the relationship of effective management (Gallardo-Vázquez and Juárez, 2022). In this regard, no particular study has been conducted to understand the role of intellectual property management and open innovation and its relationship with entrepreneurial performance.

A firm having a system and culture of gathering, exchanging, and distributing market and environmental knowledge as a result of open innovation (OI), can facilitate such firms to gain competitiveness and improve business performance (Baker and Sinkula, 1999). The paradoxical nature of the relationship between IPM and OI makes them contradictory (Bogers, 2011), as both fail to support each other (Bigliardi et al., 2020) because IPM might act as an enabler or disabler of OI (Alexy et al., 2009). Open innovation literature also highlights that for any OI accepted by the firm, there must be some OI offering from the firms to market (Huizingh, 2011). Nevertheless, firms are not equally participative in contributing to this process. Plenty of research has been conducted from the perspective that the firms adopt the OI from outside (Aarikka-Stenroos and Sandberg, 2012; Aarikka-Stenroos et al., 2014; Do, 2015), as compared to the firm selling its innovation to the market. A research work by Stanko et al. (2017) concluded that research works on OI buying are more than double in contrast with the number of articles on OI selling, indicating that the commercialization perspective has not been given due importance. Therefore, the important aspect of commercialization performance (CP) should be examined (Xiaolong et al., 2021).

A review of previous research work on OI (Van De Vrande et al., 2010; West and Bogers, 2011; West et al., 2014; Dahlander et al., 2021) discloses that it has been covered from multiple perspectives, including the significance of external sources of innovation, integration of internal and external resources, the effectiveness of openness, and the types of open, innovative activities (Cheng and Huizingh, 2014; West and Bogers, 2014; Egbetokun et al., 2019). Still, there is a gap in existing studies that do not uncover the relationship between open innovation and SME commercialization performance (CP). A few studies have focused on the effects of technology in facilitating open innovation, approaches, and applications to optimize the external networks (Dodgson et al., 2006; Huston and Sakkab, 2006; Rohrbeck et al., 2009; Hienerth et al., 2014; Santoro et al., 2019). No doubt, open innovation provides great opportunities for the business management to design new and alternative ways for business practices, but the role of open innovation must be understood with the moderating role of information communication technology (García-Sánchez and García-Sánchez, 2020). Only a few studies have examined the role of information communication technologies (ICT) that can influence the relationship between OI and commercialization performance, similarly between CP and entrepreneurial performance (EP). Additionally, no study has been conducted to address the mediating role of CP between OI and EP. Furthermore, the moderating role of information communication technology (ICT) in understanding the relationship between managing the business for commercialization and a firm’s entrepreneurial performance is also an essential theoretical gap.

Therefore, this study aims to determine the impact of intellectual property management on developing the digital economy and regional entrepreneurial activity in SMEs. It is essential to understand that this gap in the literature has led different studies in this direction to provide effective and understandable information for the business organizations and their performance. However, no study was conducted to address the above-identified gaps in the literature. This study also aims to identify to what extent the role of open innovation is critical in the performance of the business and development of the digital economy. Besides, this study also aims to define the moderating role of information communication technology in dealing with business ideas in the most suitable way to compete with large business organizations. Similarly, this study is vital to consider because it provides a detailed insight into the relationship between open innovation and entrepreneurial performance with the moderating role of information communication technology that has changed the world’s dynamics with more innovation and easy-to-access communication.

## Literature review and hypotheses development

**FIGURE 1:**
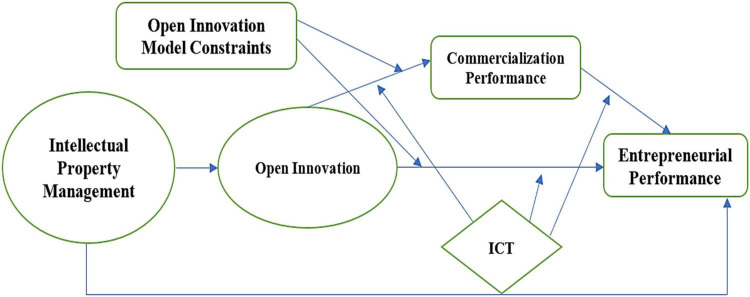
Research model. IPM, Intellectual Property Management; OI, Open Innovation; CP, Commercialization Performance; ICT, Information Communication Technology; EP, Entrepreneurial Performance; OIMC, Open Innovation Model Constraints.

In this study, the research model is developed with the help of organizational performance theory (see Figure 1). According to organizational performance theory, the organization’s performance is directly dependent on different factors contributing to this performance (Shafique and Khan, 2020; Masenya, 2022). These factors are the role of effective management, the employee’s motivation, and the environment of the working behavior (Hanly et al., 2022). The dependent variable of this study is entrepreneurial performance, a mediating variable is commercialization performance, and the independent variable is intellectual property management. Furthermore, the moderating variable of this study was open innovation model constraints. Notably, the dependent variable of this study is entrepreneurial performance. Therefore, this theory supports the research model of the study. This theory provides a detailed insight that the organizational performance leads the organization to effectively develop sustainability in the target market (Udriyah et al., 2019; Khan and Ghayas, 2022). Therefore, the consideration of this theory was more related to the understanding of the research model because the ultimate output was the same in organizational performance.

Furthermore, this theory relates to organizational performance and its relationship to different factors that support organizational performance. In this regard, more emphasis on the organization would be to be supported with different variables that are dynamic and changeable from business to business (Leroy et al., 2022). However, it is also noted that this theory was widely used in different studies that were considered adequate for developing the theoretical frameworks of those studies. Besides, this theory is implemented in different organizations to measure the performance of employees for getting the things done in the appropriate way that is best to develop the relationship between the hypotheses of the study. Chesbrough (2003) and Lichtenthaler (2010) tested the relationship between intellectual property and open innovation, but these studies did not consider entrepreneurial performance. Similarly, research conducted by Hung and Chiang (2010) and Ju et al. (2013) checked the relationship between open innovation and entrepreneurial performance and suggested checking the mediating role of commercialization performance. Therefore, the study’s theoretical framework considers the mediating role of commercialization performance.

### Relationship between intellectual property management, open innovation, and entrepreneurial performance

Intellectual property management protects the innovative ideas of business people conducting any business activity in the society (Hartono et al., 2022). It is essential to understand that protecting the business is the responsibility of the government, and the business people who are provided with the intellectual property right get to benefit from it while conducting their business activities. In this regard, according to Shaikh (2018), the role of intellectual property is the right pathway to the business organisations for the betterment of new ideas in business performance to solve society’s problems. The critical role of intellectual property laws is vital in any society because based on these laws, the moral standard of any society is set and it provides a pathway for better understanding the society (López-Arceiz et al., 2022). The business practices with intellectual property rights take the organizations to innovation because the unique ideas of the organization are protected to provide a sustainable and well-developed right to protect the unique business ideas. In this regard, the government’s responsibility is to provide legal entitlement to the business to protect the unique ideas of the businesses in a protective way to promote the culture of innovation in the organization (Thamagasorn and Pharino, 2019). Indeed, the business organizations that are provided with adequate intellectual property management support from the society provide a roadway to business for sustainable development in the society, which is critical for the sustainability and corporate social responsibility (Gryshchenko et al., 2022).

On the one hand, in the advanced and developed countries, the legal obligations related to intellectual property rights are performed for the betterment of the society because when these rules and regulations protect the unique business ideas, they lead the organizations to the way of innovation in business performance and development of the products and services (Udriyah et al., 2019). On the other hand, in the third-world countries, due to a lack of legal and procedural obligations, the business ideas are not protected with the help of intellectual property rights, which leads to the decrease in innovative ideas for the development of products and services in the organizations because they are not protected (Andrei et al., 2020). In this way, the responsibility of the management and the stakeholders is to provide legal obligation related to the intellectual property management to promote the culture of innovative and unique products and a service department that would ultimately help the business organizations in the long run. In America and Canada, the business organizations work according to the legal regulations related to intellectual property rights to understand the right of other business organizations for the better development of culture, they do not copy the unique ideas of the other people (Arraya et al., 2015; Khan and Ghayas, 2022). In this way, the business organizations can get the patent or copyright on the product or services built on the unique ideas to protect the services and products from being copied by illegal and low-category businesses. In China, being a large manufacturing and services business industry, the government of China is providing the intellectual property right to the business owners who are working to develop more practical and unique business ideas for the implementation to develop products and services for the betterment of the society by satisfying the needs of the customer with innovative products and services (Aliasghar et al., 2020). Moreover, if the business organizations fail to integrate the new and unique ideas with the intellectual property right, it would be challenging for them to develop social responsibility and provide best services to people (Ivanova-Gongne et al., 2022).

***H1.*** *There is a relationship between Intellectual Property Management and Open Innovation.*

***H2.*** *There is a relationship between Intellectual Property Management and Entrepreneurial Performance.*

### Relationship of open innovation, commercialization performance, and entrepreneurial performance

Open innovation is the process of sharing the idea with the other organizations for the innovative process to have more complicated and more advanced product development for the target market (Dorobantu et al., 2022). In this regard, according to Abdelaziz (2021), it is crucial to understand that business owners who are working in different categories of business are more flexible with the open innovation because they believe that open innovation is the only solution for managing business to enhance the experience and gain maximum profit. However, management practices of the past are not suitable for business owners to go with the concept of open innovation because it was believed that if the ideas of the business would be shared with public health, it would be difficult for the businesses to protect these ideas and there would be the copy of that product service in the market (Abushaikha et al., 2018). Moreover, over time, the market scenario was changed, and the rules and regulations related to open innovation were introduced to protect the people’s intellectual property rights. There would be a low-side compromise for copying different businesses’ extended products and services (Ivanova-Gongne et al., 2022). The more intellectual and the more public ideas need to be protected because if these ideas are shared manually with everyone, then there are chances of the breach in the patent of business organizations (Masenya, 2022).

In this regard, it is the responsibility of the business management to develop the strategy and plan for open innovation to define the product and services by providing more innovative ideas by the business. In Australia, the business organizations are working with the ideology of open innovation because it is believed that the ideology of open innovation is more suitable work to develop the new product and services for the target market to satisfy the customer to the advanced level (Andrei et al., 2020; Abdelaziz, 2021; Hameed et al., 2021). It is also believed that if the target market is protected and the customers are provided with the product and services according to their satisfaction requirements, it would be best for the businesses to perform commercialization activities in the target market. On the one hand, according to Masenya (2022), the responsibility of the business management is to ensure commercialization activities, and the ideology of open innovation is to provide the business with better product development which are beneficial for both small and large businesses. In such a case, if the management of the business organization is not playing a critical role per activity in the target market, then it would be difficult for the business organization to go with the opportunities of commercialization and enhance the target market by developing the products and services to make the standard of the expectations of the customers (Oshodin and Omoregbe, 2021; Rachmawati et al., 2022).

In countries such as India, the government is not efficiently working to provide patents or other intellectual property rights to business owners, and this demotivates the Indian entrepreneurs for not developing the product and services on the unique ideas because such kinds of ideas are not protected in the society (Ivanova-Gongne et al., 2022). Although all business organisations are working on unique ideas, it is the core value of any business to have intellectual property rights on products and services that are easy to access and easy to manage. The implementation of laws related to intellectual property rights gives confidence to the development team of a business and increases the protection of unique ideas. In Mexico, business management cannot get intellectual property rights because of the malpractices in business management, and due to such kinds of practical, unique, and innovative ideas, the Mexican business owners are not protected from the legal obligation (Abdelaziz, 2021). In this way, the decrease in open innovation happens because the ideas related to the business activities are not protected, and the unique ideas are adapted by different businesses, not ethically.

***H3.*** *There is a relationship between Open Innovation and Entrepreneurial Performance.*

***H4***. *There is a relationship between Open Innovation and Commercialization Performance.*

***H5***. *There is a relationship between Commercialization Performance and Entrepreneurial Performance.*

***H6***. *There is a mediating role of Commercialization Performance in the relationship between Open Innovation and Entrepreneurial Performance.*

### Moderating role of open innovation model constraints

Open innovation allows businesses to collaborate to provide more effective and reliable offers to each other for developing the product and services for maximum benefit with minimum resources and collective use of human capital (Ottosson and Kindström, 2016; Tiep Le and Nguyen, 2022). However, according to Flammer (2015), the management of businesses is not well-educated and well aware of the performance innovation because it is still believed that open innovation is not the appropriate solution for developing the product and services to provide in the target market. When sole proprietorship businesses carried out business operations in the past, the management took into account the efficacy of goods and services in connection to the success of the firm’s commercialization (Obermayer et al., 2021). Moreover, the development of medium and large organizations has brought a new era of business performance in the target market that has led organizations to collaborate and share ideas to get maximum profit and maximum competitive advantage in the target market. In this regard, it is crucial to understand that the business performance is not only related to the performance of the organizations, but it is the performance of the business in the same category when they are working in a collective way to develop a strategy for effective management (Andrei et al., 2020; Gryshchenko et al., 2022).

In this way, the joint and collective effort of different firms leads the businesses to the competitive advantage essential to consider in the target market because of globalism. The new globalization trends and the working of multinational organizations in different countries have challenged the local business unit in the target market for commercialization (Kato and Charoenrat, 2018). According to Abdelaziz (2021), in India, different multinational organizations are working and providing products and services to the target market, opposite to the Indian organizations that are leading the whole scenario differently. Therefore, the joint venture and collaboration between businesses are essential to consider to defeat different organizations that are providing opportunities and working in a more comfortable and reliable way in the target market to improve the business performance and enhance the profit with commercialization activities (Udriyah et al., 2019; Oshodin and Omoregbe, 2021). The responsibility of the business management is to understand the changing dynamics of the target market and develop a strategy with the idea of sharing open innovation with businesses in America to develop a competitive advantage in the most prominent target market (López-Arceiz et al., 2022). The way of open innovation would provide an opportunity for entrepreneurial activities and increase the business performance of the business organization to work in a more competitive era. According to Khan and Ghayas (2022), organizations that are working on improving business practices are more reluctant to provide services to people in the best and most effective way.

The information communication technology system connects the organizations in China and America to share all the information by working collectively to boost the economy and provide more reliable and effective products in the target market that would be beneficial for the people in the long term (Abushaikha et al., 2018; Yasa et al., 2020). For businesses that are not working on the modern trend and not utilizing information communication as a system to integrate all the related things, it would be challenging for the business organization to enhance the management experience by interacting with other organizations. In this regard, the responsibility of the business management is to provide the best alternative ways and determine all kinds of critical factors that are contributing to effective management for the commercialization performance and the business performance that the innovative leaders manage (Arraya et al., 2015; Andrei et al., 2020; López-Arceiz et al., 2022). Therefore, the responsibility of the government and other stakeholders is to provide opportunities to the businesses to communicate with each other and share the ideas for open innovation to develop different strategies and prosper in the business performance in the target market for commercialization performance.

***H7.*** *There is a moderating role of Open Innovation Model Constraints on the relationship between Open Innovation and Entrepreneurial Performance.*

***H8***. *There is a moderating role of Open Innovation Model Constraints on the relationship between Open Innovation and Commercialization Performance.*

### Moderating role of information communication technology

Information communication technology is enhancing the experience of the world in business performance and developing sustainability with the influential factors of globalization (Niyaz et al., 2015). Globalization is a vital driver that relates it to easier-to-access information systems, that is, information communication technology. It is essential to understand that information communication technology has changed the world’s dynamics and has provided more reliable and understandable business opportunities to the different business organizations worldwide (Khan and Ghayas, 2022; Xu et al., 2022). However, not only the information communication technology but the business management is also responsible for integrating the business world from the different countries and performance that would be beneficial for the target market in an effective way (Andrei et al., 2020). In this regard, the performance of different entrepreneurship is dependent on the performance of the business organization in the target market with the help of information communication technology to enhance the business experience. Indeed, Xu et al. (2022) state that the responsibility of business management in work according to the packing of open innovation in business performance to increase commercialization that would lead the organisation to advanced performance and understandable opportunities to integrate all operational departments in similar business activities. Moreover, it is also essential to understand that if the business management is not provided with the right opportunities with the help of open innovation, then that decline in commercialization performance would be a restriction on business performance (Arfi et al., 2018).

Advanced businesses are working on the concept of innovation ideal and open innovation to enhance the business practices for commercialization, but in such a kind of business organization, the role of information communication technology is essential. It is a fact that information communication technology provides a way for the business organization to develop a better understanding of the organization with innovative ideas to become a leader in the target market (Zeb et al., 2021). Remarkably, the business organization working on profiling benefits society on corporate social responsibility guidelines. These organizations are more innovative in their product and services, and the integration of information communication technology led them to a more advanced level and have more benefits in the target market (Martínez et al., 2019; Khan and Ghayas, 2022). Similarly, according to the study by López-Cabarcos et al. (2022), business organizations are using information communication technology to improve business practices in Canada because, with such kinds of practices, the reliability and experience of business organization management would be more customer-oriented to develop different kinds of strategies for the improvement in the performance of entrepreneurship business.

Moreover, in backward countries, business organizations are not provided with opportunities for information communication technology to enhance business performance with more liable and understandable strategies for the development of businesses (Rachmawati et al., 2022). As a result, these countries’ small and medium organizations are left behind and not provided with the right opportunity to improve their business practices and provide the appropriate solution to work in the guidelines of corporate social responsibilities to improve the standard of products and services. The management considers that more effective corporate social responsibility would help businesses to develop a more reliable and understandable public image in the target market for the benefit of the business organization to the advancement of the organization (Hida and Dewi, 2021).

Information communication technology plays a critical role in developing a modern organization that works worldwide over the Internet. It is because, with the help of information communication technology, more emphasis on the development of communication systems has provided a unique and alternative way for the organization to communicate with the customer and provide innovative business services or products to the target market on time. Similarly, the business organization is connected to the joint development of strategies for getting a competitive advantage in the target market with the help of collaboration between the management of the different organizations (Taj et al., 2019; Wang and Liu, 2019). However, in this regard, the role of information communication technology has increased because it provides a way to open innovation to communicate all the ideas with different organizations worldwide and connect these organizations to work as a joint venture. No doubt, the purpose of every organization is to develop comparatively better in the target market than the other organization that is enhancing the experience of the management by sharing the collective ideas to develop a competitive advantage (Anderson et al., 2018).

***H9.*** *Information Communication Technology’s moderating role is in the relationship between Open Innovation and Entrepreneurial Performance.*

***H10.*** *There is a moderating role of Information Communication Technology in the relationship between Open Innovation and Commercialization Performance.*

***H11***. *Information Communication Technology’s moderating role in the relationship between Commercialization Performance and Entrepreneurial Performance.*

## Methodology

### Prepare questionnaire

In this section of the study, the development of the questionnaire is discussed. The questionnaire for this study was divided into two different parts. The first part was related to the respondents’ demographic information. However, the second part consisted of the scale items taken from different creditable studies to measure the relationship between different variables and test the hypotheses. The questionnaire was developed on a five-point Likert scale because it is the most suitable process to collect the data from the respondents, according to the study by Nawaz et al. (2022). In this regard, four scale items for intellectual property management were taken from the study by Singh and Misra (2021). Similarly, to measure open innovation, four scale items and to measure open innovation model constraints, three scale items were taken from the study by Hosseini et al. (2018). Also, to measure commercialization performance, three scale items and entrepreneurial performance were taken from the study of Rumengan et al. (2018). Finally, four scale items to measure information communication technology were taken from the study by Hameed et al. (2018).

### Data collection process

The population of this study is the SMEs of China because China has more than 40 million, and almost 5 million SMEs are added each year (Statista, 2022). Therefore, for this study, the data from management of SMEs in 30 provinces (including Heilongjiang, Hubei, Guangdong, Beijing, and Shanghai) in China, because these were the study’s respondents, was collected. First, the firms listed on the SME and Growth Enterprise Board in China were compiled. And then, using a simple random sampling technique, 700 questionnaires were sent to the management of sample companies. A detailed study introduction was provided to the respondents, and a questionnaire to collect the data. For it, questionnaires were provided to the respondent with the paid return envelope. Second, the respondents were asked to provide an impersonal response to the questionnaire to contribute to the study.

Moreover, the researcher’s email was also provided to the respondents to deal with any queries. In this regard, all the respondents’ queries were addressed effectively to provide more reliable insight into the study. However, only 577 questionnaires were collected from the respondents to proceed with the study. Also, the received questionnaire was analyzed with the help of research experts, and 530 questionnaires were selected for the final data analysis of the study. In this way, the survey questionnaire was considered more attractive and more reliable for this study because it is a time-saving and understandable method for the respondents to respond in the best way for the study.

## Findings

In this study section, confirmatory factor analysis was done to explain the structure of the variables and scale items (see Table 1). In this regard, the items with low factor loadings of 0.40 were rejected. Furthermore, four items for intellectual property management, four for open innovation, three for commercialization performance, four for information communication technology, three for open innovation model constraints, and three for entrepreneurial performance were taken for this study that has significant factor loadings. In this way, the confirmatory factor analysis was used to measure the reliability and validity of the scale items used in this study.

**TABLE 1:** Results of confirmatory factor analysis.

Construct	Description	Alpha	Standardized factor loadings
Intellectual property management	Intellectual property management reliable.	0.81	0.824
	Intellectual property management helps business performance.	0.843	
	Intellectual property management is suitable for small and medium businesses.	0.784	
	Intellectual property is protected for business performance.	0.759	
Open innovation	Open innovation enhances business performance.	0.84	0.804
	Open innovation develops a competitive advantage	0.828	
	Open innovation helps CSR	0.795	
	Open innovation promotes new ideas.		0.671
Commercialization performance	Open innovation increases commercialization performance	0.82	0.752
	Intellectual property management boosts commercialization performance	0.867	
	Commercialization performance protects small businesses	0.848	
Information communication technology	Information communication technology helps in business performance	0.78	0.600
	Information communication technology provides sustainable business practices	0.818	
	Information communication technology develops a competitive advantage	0.819	
	Information communication technology is best for digital economy	0.810	
Open innovation management constraints	Open innovation management face the issue of finance.	0.816	0.848
	Open innovation management face the issue of research and development department.	0.861	
	Business practices with open innovation are successful, but it is not easy process.	0.782	
Entrepreneurial performance	Entrepreneurial performance develops digital economy	0.79	0.743
	Entrepreneurial performance boosts the economy	0.877	
	Entrepreneurial performance is improved business performance	0.819	

### Measurement model

**FIGURE 2:**
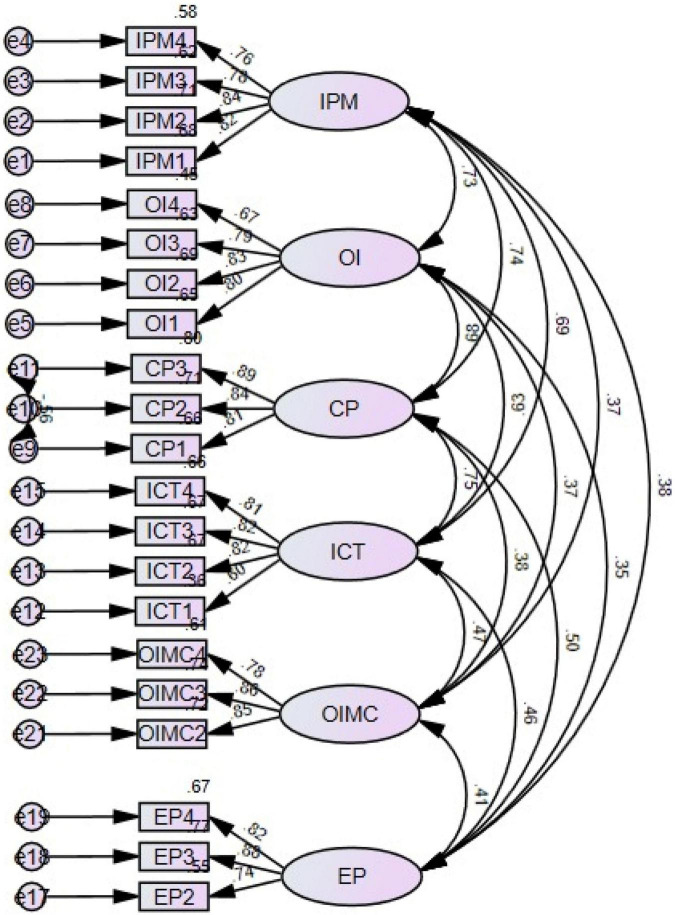
Measurement model. IPM, Intellectual Property Management; OI, Open Innovation; CP, Commercialization Performance; ICT, Information Communication Technology; EP, Entrepreneurial Performance; OIMC, Open Innovation Model Constraints.

Furthermore, a discriminant validity test was conducted to measure the irrelativity of the measure used for a single construct. The AVE of each construct must be greater than the squared correlation between the construct with other constructs. According to the results presented in Table 2, the squared multiple correlations provide a clear discriminant validity. In Figure 2, the visual description of the measurement model is presented.

**TABLE 2:** Reliability, validity statics, and correlations.

	CR	AVE	MSV	MaxR(H)	IPM	OI	CP	ICT	EP	OIMC
IPM	0.879	0.645	0.549	0.883	**0.803**					
OI	0.858	0.604	0.537	0.867	0.733***	**0.777**				
CP	0.885	0.72	0.569	0.892	0.741***	0.675***	**0.848**			
ICT	0.849	0.589	0.569	0.867	0.687***	0.632***	0.754***	**0.767**		
EP	0.855	0.664	0.246	0.868	0.376***	0.354***	0.496***	0.458***	**0.815**	
OIMC	0.87	0.691	0.223	0.875	0.374***	0.365***	0.376***	0.473***	0.409***	**0.831**

IPM, Intellectual Property Management; OI, Open Innovation; CP, Commercialization Performance; ICT, Information Communication Technology; EP, Entrepreneurial Performance; OIMC, Open Innovation Model Constraints. *p-value < 0.05; **p-value < 0.01; ***p-value < 0.001. Bold values are discriminant validity.

Similarly, this study analyzed the measurement model fit by evaluating the root mean square of approximation, absolute fit measures, standardized root mean square residual, comparative fit index, normed fit index, and adjusted goodness of fit. Similarly, the recommended threshold was achieved for evaluating the model (see Table 3).

**TABLE 3:** Fit indicates of CFA model.

Measure	Abbr.	Scores
Chi-square/df (CMIN/DF)	2/df	2.105
Comparative fit index	CFI	0.9
The normed fit index	NFI	0.89
Goodness of fit	GFI	0.79
Adjusted goodness of fit	AGFI	0.81
Root mean square residual	RMR	0.08
Standardized root mean square residual	SRMR	0.07
Root mean-square error of approximation	RMSEA	0.07

### Structural model

**FIGURE 3:**
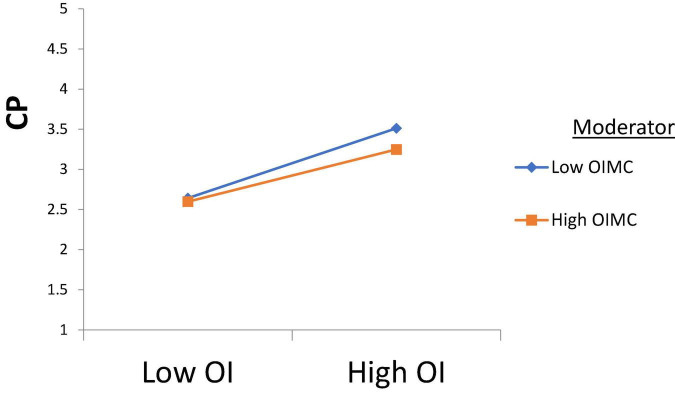
Moderation effect 1. OI, Open Innovation; CP, Commercialization Performance; OIMC, Open Innovation Model Constraints.

**FIGURE 4:**
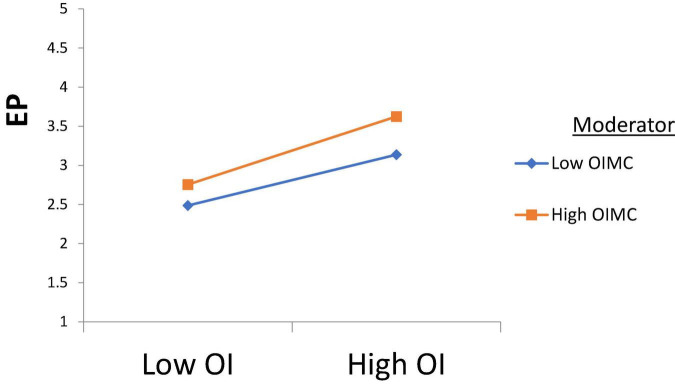
Moderation effect 2. OI, Open Innovation; EP, Entrepreneurial Performance; OIMC, Open Innovation Model Constraints.

**FIGURE 5:**
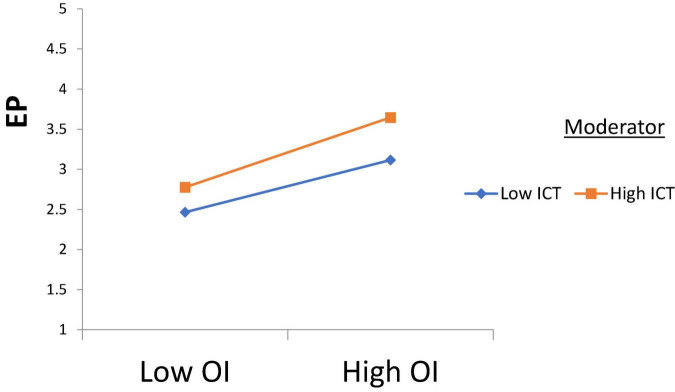
Moderation effect 3. OI, Open Innovation; ICT, Information Communication Technology; EP, Entrepreneurial Performance.

**FIGURE 6:**
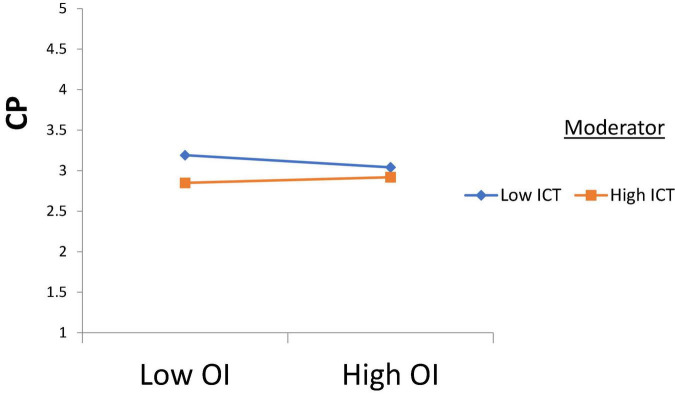
Moderation effect 4. OI, Open Innovation; ICT, Information Communication Technology; CP, Commercialization Performance.

**FIGURE 7:**
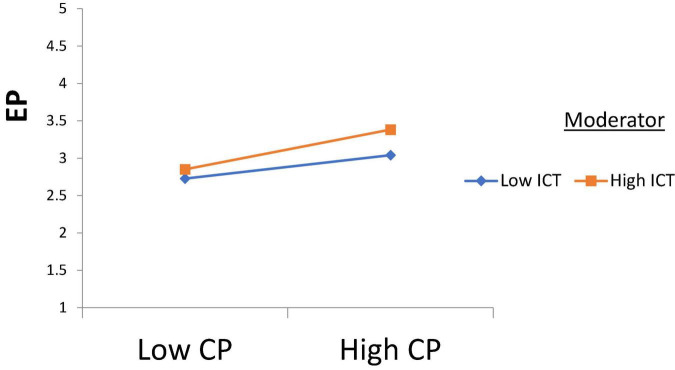
Moderation effect 5. CP, Commercialization Performance; ICT, Information Communication Technology; EP, Entrepreneurial Performance.

H1 was tested, and according to the results, Intellectual Property Management significantly affects Open Innovation (β = 0.250, *t* = 2.710); thus, H1 is accepted. H2 was tested, and according to the results, Intellectual Property Management has a significant effect on Entrepreneurial Performance (β = 0.170, *t* = 3.170), H2 is supported. H3 was tested, and the results revealed that Open Innovation has a significant effect on Entrepreneurial Performance (β = 0.189, *t* = 2.870); thus, H3 is approved. H4 was tested to check its significance, and according to the results, Open Innovation has a significant effect on Commercialization Performance (β = 0.380, *t* = 2.261); thus, H4 is accepted. Furthermore, H5 was tested and the results indicated that Commercialization Performance has a significant effect on Entrepreneurial Performance (β = 0.281, *t* = 2.912), and H5 is accepted (see Table 4). According to the results of H6, Commercialization Performance mediates the relationship between Open Innovation and Entrepreneurial Performance (β = 0.107, *t* = 2.435); therefore, H6 is accepted (see Table 5). Furthermore, according to the results, Open Innovation Model Constraints do not moderate the relationship between Open Innovation and Entrepreneurial Performance (β = 0.077, *t* = 0.780). Therefore, H7 is rejected (see Figure 3). Also, according to the results, Open Innovation Model Constraints moderates the relationship between Open Innovation and Commercialization Performance (β = 0.210, *t* = 2.821). Hence H8 is accepted (see Figure 4). Similarly, according to the results, Information Communication Technology moderates the relationship between Open Innovation and Entrepreneurial Performance (β = 0.198, *t* = 2.791). Hence H9 is accepted (see Figure 5). Moreover, according to the results, Information Communication Technology does not moderate the relationship between Open Innovation and Commercialization Performance (β = 0.076, *t* = 0.071). Hence H10 is rejected (see Figure 6). Finally, according to the results, Information Communication Technology moderates the relationship between Commercialization Performance and Entrepreneurial Performance (β = 0.211, *t* = 2.612). Hence H11 is accepted (see Figure 7). The results of the moderation hypotheses are available in Table 6.

**TABLE 4:** Standardized path coefficient.

Hypotheses	Relationship	Beta	*T*-value	Status
H1	Direct	0.250	2.710	Accepted
H2	Direct	0.170	3.170	Accepted
H3	Direct	0.189	2.870	Accepted
H4	Direct	0.380	2.261	Accepted
H5	Direct	0.281	2.912	Accepted

**TABLE 5:** Mediation results.

Hypotheses	Relationship	Beta	*T*-value	Status
H6	Mediation	0.191	2.721	Accepted

**TABLE 6:** Moderation results.

Hypotheses	Relationship	Beta	*T*-value	Status
H7	Moderation	0.077	0.780	Rejected
H8	Moderation	0.210	2.821	Accepted
H9	Moderation	0.198	2.791	Accepted
H10	Moderation	0.076	0.071	Rejected
H11	Moderation	0.211	2.612	Accepted

## Discussion

This manuscript contributes significantly to the current body of literature by suggesting a novel framework addressing essential intellectual property management strategies and enhancing entrepreneurial performance. According to H1 and H2, there is a significant relationship between intellectual property management, open innovation, and entrepreneurial performance. IPM is a crucial factor in facilitating the innovation process. SMEs where a sufficient system of IPM is established, ensuring the protection of novel ideas from its intellectual capital, share the innovation information openly to compete with other businesses. As Lichtenthaler (2010) discussed, open innovation only depends on the firm’s decision. It is critical to understand that in such kind of business practices, with the help of intellectual property management, the critical success factor increased due to open innovation, and the competitive advantage of the organization was achieved in the target market (Thompson et al., 2014; Singh and Misra, 2021; Mathafena and Msimango-Galawe, 2022). Therefore, it is the responsibility of the management to ensure that all the measures are taken to manage and protect the open innovation in the target market (Skordoulis et al., 2020).

According to the results of H3 and H4, there is a significant relationship between open innovation, commercialization performance, and entrepreneurial performance. The results are similar to the study by Chesbrough (2003). The role of open innovation in achieving entrepreneurial performance was reaffirmed, as in earlier research studies (Wang, 2008; Avalos-Quispe and Hernández-Simón, 2019), which have shown that open innovation influences corporate performance. Similarly, according to the study by Cascella et al. (2022), the organization gets a competitive advantage at the target market because innovative ideas are shared and implemented practically. The more information shared between the management, the more product or service development procedure would be continued—commercialization results from the open innovation.

According to the results of H5, there is a significant relationship between commercialization performance and entrepreneurial performance. The findings support Kim and Kim’s (2018) hypothesis that putting more focus on business development initiatives enables companies to examine, define, and integrate novel inputs and ideas into current processes, as well as offer new solutions that may (indirectly) further enhance the firm’s commercialization performance and results in more revenue and good reputation of the company. The ability of enterprises to delink existing intellectual capital from established product–market pairings and relink them to new product lines and niches is critical for the marketing of novel goods (van Hemert et al., 2013). It is similar to the previous findings that it is boosting the economy of China in the world, as highlighted in the studies of Asadi et al. (2022). In this regard, open innovation is the key to success in the entrepreneurial and management performance for the successful business performance to develop a competitive advantage in the target market. The opportunity for open innovation must be accepted by emerging businesses to lead the organization constructively. It is critical to understand that commercialization performance has increased in the digital economy because this has led small and medium businesses to the next level of achievements (Avotra et al., 2021; Yingfei et al., 2021). In this regard, the responsibility of management is not limited to improving open innovation and intellectual property management. However, at the same time, it must be done to increase the entrepreneurial and commercialization performance in the target market (Dorobantu et al., 2022). Indeed, if the government policies are better for the commercialization performance and are supported by open innovation and intellectual property management. As a result, more emphasis would be placed on developing entrepreneurial performance in the target market (Khan and Ghayas, 2022; Putri and Honggare, 2022). The organization should consider the effectiveness of commercialization performance for the advancement of business in large markets as discussed in the study by Anjum et al. (2020). Therefore, more emphasis would be on the management to deal with all kinds of obstacles in the way of successful business performance.

According to the results of H6, there is a significant mediating role of commercialization performance in the relationship between open innovation management and entrepreneurial performance. No doubt, the business practices in the global world are different from the traditional business practices because now the economy has become a digital economy. The responsibility of the management is to provide the best alternative ways for improving the business practices for the betterment of the economy. Commercialization performance is critical to the success of the business because if a business is only focused on acquiring the innovation for its internal use and not sharing it with others, its performance may face challenges, as discussed in the study by Khan and Ghayas (2022), and it would be challenging for the management to generate more revenue (Clauß et al., 2022). Therefore, according to the study by Mathafena and Msimango-Galawe (2022), the responsibility of management is to improve the business performance, provide more alternative ways for getting better results, and provide better opportunities to enhance business practices. According to the results of H7, there is no significant moderation of intellectual property model constraints between open innovation management and entrepreneurial performance. It is to be noted that firms that initiated open innovation management tend to perform better because they have gradually overcome all the constraints. In this regard, the responsibility of the management is only to ensure that open innovation management is working best without any external interference, and it provides the best practices for improving the business performance (Murad et al., 2022). Therefore, more emphasis would be on the performance of the business to boost the digital economy with open innovation initiative.

According to the results of H8, there is a significant moderating role of the Open innovation model constraint between open innovation and commercialization performance. In this regard, according to López-Cabarcos et al. (2022), it is crucial to understand that for the performance of the economy in any country, there is a critical role of open innovation if it is done under the guidelines of the open innovation model. However, the organizations that fail to design an effective open innovation model have to face constraints, and the concept of open innovation is lame for them (Khan and Ghayas, 2022; Putri and Honggare, 2022). Despite this, the management must work to improve the practices for getting the things done in more innovative ways by providing the opportunities to get protection for the innovative ideas so that it can share them with the outside world (Dorobantu et al., 2022). Such collaboration will enhance the firm’s commercialization performance.

According to the results of H9, information communication technology has a significant moderating role in the relationship between open innovation and entrepreneurial performance. If the business follows an open innovation management policy, then a good information communication technology infrastructure will further enhance business performance, and the findings are aligned with that of Ju et al. (2013). In this way, the performance of business activities would be increased to an advanced level. Although developing the ICT infrastructure firm’s cost will increase, the firm’s performance will eventually improve.

According to the results of H10, there is no moderation of information communication technology between open innovation and commercialization performance. These results do not match the study of Skordoulis et al. (2020). It is because if the management provides open innovation for the effectiveness in business performance and commercialization performance, then there would be no need for information communication technology to a greater extent. However, if the commercialization performance is not improving effectively and it is facing hurdles, then the critical advancement in open innovation would lead the business organization to productivity. Therefore, information communication technology is undoubtedly a way for successful globalism and business performance but is not a critical success factor in commercialization performance (Xu et al., 2022). According to the results of H11, information communication technology has a significant moderating role in the relationship between commercialization performance and entrepreneurial performance. Indeed, for business performance, information communication technology matters a lot because it is a factor in communication with the clients and the other business (Hu and Huang, 2022; Putri and Honggare, 2022). Therefore, the management best consider information communication technology as a competent and reliable tool for business management to ensure productivity and increment in the revenue of the business. In this way, more emphasis would be on the business development, leading the organization to the best sustainable goals.

## Conclusion

Our study developed a framework to see how IPM is associated with entrepreneurial performance. This study provides information about the open innovation initiatives taken by the management in different SMEs and its outcome. Indeed, the organizations are working with the open innovation approach to balancing the challenges offered by digitalization and globalization. However, it is to be ensured that SMEs devise appropriate systems of intellectual property management to avoid any copy of products or services by any other company in an unethical way (López-Arceiz et al., 2022). Our findings suggest that the more effort companies exert into the intellectual property management systems, the more open flow of innovation would be in an organization. If the open innovation flows inward, the employees will enhance their skills. If the open innovation is outward, the structure of collaboration among the business partners would be increased (Udriyah et al., 2019), which will give SMEs a competitive advantage and take measures to commercialize their innovations. Therefore, the management’s responsibility is to design the organization’s systems and culture in ways that support enhancing the practices for the collective benefits and more adaptable sharing ideas through open innovation (Hu and Huang, 2022). No doubt, management practices are a difficult task to get better results as an output of the improved business performance. Therefore, for improved entrepreneurial performance, business management needs to opt open innovation philosophy.

When SMEs have opted for the open innovation strategy, then barriers to improved performance mean very little to them. They keep working on innovative ideas for continuously improved performance (Avalos-Quispe and Hernández-Simón, 2019). However, these open innovation model constraints hinder commercialization performance. Furthermore, the influencing role of information communication technology was also tested. SMEs following inward open innovation through IPM and outward OI through its commercialization are influenced by ICT systems it has. Firms with high ICT systems indicated more performance in the commercialization of innovation (López-Arceiz et al., 2022). Undoubtedly, information technology has changed the dynamics of the world by providing a way of communication with businesses to improve products and development by sharing ideas (Khan and Ghayas, 2022). In this regard, according to López-Arceiz et al. (2022), the role of information technology is critical to integrate unique ideal resources by different organizations to have both productivity and more emphasis on the development of different strategies for the target market.

### Theoretical implications

This study provides significant theoretical implications for the entrepreneurial performance of SMEs. In this regard, this study is significant as it provides a detailed insight into the relationship of different variables taken in the study’s research model. Indeed, several studies were conducted on entrepreneurial performance, but no particular study was conducted to understand the relationship between intellectual property management, open innovation, and entrepreneurial performance to boost the digital economy. In this way, this study provides a significant relationship between these variables in the context of SMEs, and further, it determines the relationship of moderating variables that were not discussed by any earlier study. First, this study provides a significant relationship of information communication technology as a moderator in the relationship between open innovation and commercialization performance and the relationship between commercialization performance and entrepreneurial performance.

Furthermore, the moderating role of information communication technology is also presented in the relationship between open innovation and entrepreneurial performance. Second, this study contributes to the literature by demonstrating the mediating role of open innovation model constructs in the relationship between open innovation and entrepreneurial performance in China and between the relationship between open innovation and commercialization, which was not discussed by any of the earlier studies. Therefore, this worthy contribution to the literature would enhance the experience of the management of SMEs working in China for better productivity and a better advantage in the entrepreneurial performance to boost and contribute to the digital economy. Moreover, this theoretical contribution would provide a relationship between different variables, and future studies related to entrepreneurial performance would benefit from it.

### Practical and managerial implications

This study also provides significant practical contributions to the impact of intellectual property protection on the development of the digital economy and regional entrepreneurial activity. It is essential to understand that no earlier study has discussed the role of these variables in the context of SMEs and entrepreneurial performance in the digital economy. In this regard, this study provides detailed guidelines to improve commercialization performance and entrepreneurial performance by taking practical measurements. This study demonstrates that there is a critical role of intellectual property management in developing SMEs with a joint effort with innovative ideas to increase entrepreneurial performance. In this way, the findings and conclusion of this study would help the stakeholders of the SMEs to engage all the management practices in a way that would be beneficial for the organizations to establish long-term goals and develop policies to accomplish those goals effectively. The more practical approach to business performance would lead the digital economy to an advanced level with the help of information communication technology. Second, this study highlights that if the organization works in a joint venture by sharing the ideas with open innovation, then more improvement in the management practices would lead the organization to a competitive advantage. However, the critical role of intellectual property management must be considered critical in business practices because it would provide guidelines to develop the strategies effectively. If there is any violation of intellectual property management, the stakeholders will react more effectively. Furthermore, this study highlights that the business managers should ensure the collaboration between SMEs because it is the best approach to utilize each resource, including open innovation ideas for the betterment of the business performance. Moreover, the responsibility of the management is to ensure all the procedure is conducted effectively to achieve sustainability in entrepreneurial performance with the help of information communication technology.

## Limitations and future directions

This study was limited to understanding the impact of intellectual property protection on developing the digital economy and regional entrepreneurial activity in the context of SMEs. This study has used information communication technology and open innovation model constraints as the moderating variable to determine their effect on entrepreneurial performance and commercialization performance. However, during the literature review, it was identified that several other factors could also moderate the relationship between open innovation of entrepreneurial performance. Therefore, future studies should understand the influencing role of innovation adoption on the relationship between open innovation and entrepreneurial performance. Similarly, the mediating role of effective management must be determined to understand the relationship between intellectual property management and SME commercialization performance. In this way, this contribution would enhance the experience of the management of SMEs to the advanced level for the development of business ideas, and this worthy contribution would effectively contribute to the literature.

## Data availability statement

The original contributions presented in the study are included in the article/supplementary material, further inquiries can be directed to the corresponding author.

## Ethics statement

The studies involving human participants were reviewed and approved by the Tongji University, China. The patients/participants provided their written informed consent to participate in this study. The study was conducted in accordance with the Declaration of Helsinki.

## Author contributions

HC conceived, designed the concept, collected the data, and wrote the manuscript. The author read and agreed to the published version of the manuscript.

## Conflict of interest

The author declares that the research was conducted in the absence of any commercial or financial relationships that could be construed as a potential conflict of interest.

## Publisher’s note

All claims expressed in this article are solely those of the authors and do not necessarily represent those of their affiliated organizations, or those of the publisher, the editors and the reviewers. Any product that may be evaluated in this article, or claim that may be made by its manufacturer, is not guaranteed or endorsed by the publisher.
